# Effects of Lighting, Liquid Color, and Drink Container Type on Volume Perception

**DOI:** 10.1177/2041669519880916

**Published:** 2019-10-10

**Authors:** Yi-Lang Chen, Yi-Chien Lee

**Affiliations:** Department of Industrial Engineering and Management, Ming Chi University of Technology, New Taipei; Department of Industrial Design, Chang Gung University, Taoyuan City; Department of Industrial Engineering and Management, Ming Chi University of Technology, New Taipei

**Keywords:** volume perception, proportionate size, elongation, lighting, liquid color

## Abstract

This study explored the effects of geometric features (i.e., proportionate size and elongation) of tumblers and goblets on volume perception under different lighting environments and with different colors of liquid. Sixty individuals (30 men and 30 women) participated in an experiment that demonstrated volume perception of a specified amount (100 ml or 200 ml, depending on the container) by pouring water or red wine into different pairs of glasses. The results revealed the goblet and tumbler pairs in both the proportionate-size and elongation groups produced significant effects on overall volume perception; by contrast, the effects of lighting and liquid color were only observed in specific groups. The geometric features of the glasses yielded inconsistent results for different pairs of glasses; these dissimilar results may have been caused by differences in visual cues (glass rim or height) affecting volume perception under different experimental settings. In addition, this study revealed that men underestimated the volume more than women did and thus poured more liquid into the glasses. In practical application, these study results should be considered in conjunction with the context and purpose of drink and container selection to understand the commensurate illusory effects.

## Introduction

Visual illusions have received increasing attention despite a lack of practical applications in the past. Recently, visual illusions have been applied to daily life; product design, such as for clothing, tableware, and marketing; and in clinical practice, such as for weight loss diets and alcohol limitation regimens. A study revealed that overweight children who participated in fitness camps drank 88% more juice when dining because they underestimated the capacity of short, wide glasses (Wansink & Van Ittersum, 2003), a phenomenon that was also observed among adults in fitness camps. In bars, 20% to 30% more alcohol was poured in short, wide glasses than in tall, slender glasses, and this is unaffected by the experience of the pourer (Wansink & Van Ittersum, 2005). Therefore, Wansink (2010) proposed the mindless eating concept in which people make decisions about the amount of their food and drink intake based on visual cues rather than by actual consumption. Vision plays the most crucial role during consumer decision-making (Fenko, Schifferstein, & Hekkert, 2010) because sight forms 80% of all perceptions, indicating that the human eye is the most essential sensory organ for object identification.

Studies on the relationship between container features and volume perception have produced different results. Piaget (1968) studied container elongation and discovered that children often judge volumetric capacity by the height of containers, ignoring the cup rim’s diameter. Raghubir and Krishna (1999) reached a similar conclusion with their study on tall, slender glasses and short, wide glasses. Attwood, Scott-Samuel, Stothart, and Munafò (2012) discovered that research participants’ misunderstanding of the midpoint of a curved glass was higher than that of a straight glass, indicating that irregular shapes complicate perceptual judgment. However, Chen and Shi (2017) obtained results opposite those of Wansink and Van Ittersum (2003) in experiments that tested the perceived capacity of water in short, wide glasses. Troy et al. (2018) also demonstrated that the shape of glasses influenced the pouring accuracy of liquid and found that pouring in tulip and curved glasses was less accurate than in straight glasses, possibly due to the height of liquid within the glass and volume changing in a nonlinear relationship.

The contradictory results among studies may be partially explained by the finding from the study of Szocs and Lefebvre (2017), who found that when viewed from a downward angle, participants perceived the portion as larger when it was presented horizontally. They concluded that when individuals view a plate of food at a downward angle (e.g., when seated at a dining table), the surface area is easier to encode than the height dimension and therefore is used as a heuristic for size. Thus, Chen, Lee, Lee, and Chen (2017) conducted a water-pouring experiment from different viewing angles and verified that the previous contradictions were indeed related to viewing angles. Wansink and Van Ittersum (2003) conducted an experiment in which participants held a glass in their hands at a viewing angle of approximately 0° or 30°. Conversely, the aforementioned experiment by Chen and Shi (2017) was performed in a seated position that produced a viewing angle of approximately 60° and shifted the participant gaze from the glass height to rim diameter, resulting in a different volume perception.

In addition, Wansink and Van Ittersum (2010) used large and small spoons to simulate the use of liquid medicine with children and discovered that variant spoon sizes caused dosage deviations. Van Ittersum and Wansink (2012) experimented with ladling soup into bowls and discovered that research participants had 8.2% less soup in a small bowl than in a standard size bowl and 9.9% more soup in a large bowl, indicating that a larger bowl size may lead to overeating. Pechey et al. (2015) applied this concept to an experiment with glasses and discovered that smaller glasses were perceived as containing more liquid than larger ones. They concluded that both drinking speed and overall consumption would decrease by serving standard portions in smaller glasses. Chen et al. (2017) also found that participants poured more water into large glasses than into small ones when viewing the process at a downward angle.

Most people are unaware that environmental factors affect volume perception (Wansink, 2004). By comparing the environmental settings of formal meals (e.g., the use of ceramic plates, glasses, silverware, and cloth napkins at a table) and snacks (e.g., the use of paper plates and napkins, plastic cups, and no utensils), Shimizu, Payne, and Wansink (2010) discovered that participants in the formal meal setting had a food intake that was 27.9% more than those in the snacks setting. Wansink and Van Ittersum (2012) studied fast food restaurants and discovered that a conventional fast food restaurant environment in the United States (e.g., bright lights and noisy music) increased food intake among customers; by contrast, indirect lighting and soft music could reduce participants’ calorie intake and improve consumer satisfaction.

In addition to holding wine, wine glasses maintain aesthetics, enhance taste, and influence volume perception (Cliff, 2001). Under the Western food culture influence, goblet usage has become common in Taiwan for drinks such as juice, in addition to wine. However, relevant research related to glass shape and volume perception has primarily focused on cylindrical glasses and rarely on goblets. Therefore, this study conducted a pouring experiment with water and red wine to understand the effects of environmental lighting and liquid color on the volume perception of geometric features (i.e., proportionate size and elongation) of different container types (i.e., tumblers and goblets).

## Methods

### Participants

Thirty male and 30 female participants aged 18 to 25 years were recruited, with average ages of 22.0 ± 2.1 and 22.3 ± 1.5 years for men and women, respectively. Each participant had no visual impairments such as color blindness or color weakness, and their normal vision or visual acuity after correction was normal.

### Experimental Containers

**Figure 1.**
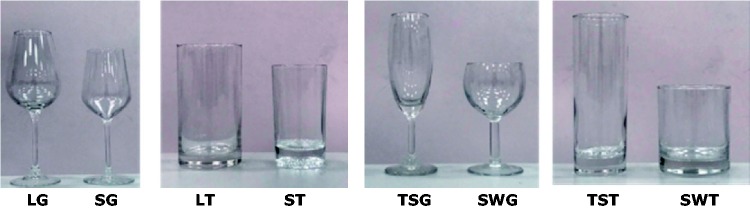
Four groups of goblets and tumblers with different sizes and elongations. LG = large goblet; SG = small goblet; LT = large tumbler; ST = small tumbler; TSG = tall, slender goblet; SWG = short, wide goblet; TST = tall, slender tumbler; SWT = short, wide tumbler.

Figure 1 presents the tumblers and goblets used in this study, which were all common containers purchased from a hypermarket to avoid complications from participants being unfamiliar with the glass type. In the proportionate-size group, the glass height–diameter ratio was identical, and the maximum capacity ratios of the small and large glasses were 50% and 70% for tumblers and goblets, respectively. In this study, the maximum capacity ratio was defined as the ratio of the maximum amount of liquid the small glass could contain to the large glass. In the elongation group, which included tall, slender glasses and short, wide glasses, the maximum capacities were identical. The capacity of all experimental glasses ranged from 150 ml (small tumbler) to 360 ml (large goblet), and the experiment required that an amount of liquid poured into the glasses between 37% and 80% of their maximum capacity. Table 1 displays the characteristics of the glasses used in the study.

**Table 1.** Maximum and Poured Volumes for Glasses With Various Characteristics.

Variables	Containers	Characteristics	Maximum volumes (ml)	Poured volumes (ml)	Percentage of the maximum volumes for pouring
Proportionate size	Tumbler	Large	300	100	37
		Small	150	100	67
	Goblet	Large	360	200	56
		Small	250	200	80
Elongation	Tumbler	Tall, slender	280	200	71
		Short, wide	280	200	71
	Goblet	Tall, slender	180	100	56
		Short, wide	180	100	56

### Experimental Design and Procedure

The participants poured water and red wine into different tumblers and goblets under two lighting levels, and those amounts were recorded and compared with target amounts. The two independent variables were the tumbler and goblet containers, each divided into proportional sizes (large vs. small) and elongation (tall and slender vs. short and wide) based on their geometric features (Figure 1). The two lighting levels were based on the Wansink and Van Ittersum’s (2012) experiment in which the illumination for the bright environment was set to 1,000 lux, whereas the dark environment simulated that of a bar with illumination at 80 lux. The two liquid colors were transparent (water) and dark red (red wine). Because each participant poured a designated amount of water (100 or 200 ml) into specified pairs of glasses, 480 volume-perception data items were collected (60 Participants × 2 Glass Pairs [either size or elongation] × 2 Lightings × 2 Liquid Colors) for each test condition including two containers and two geometric features. In this study, a counterbalanced order was adopted for each experimental pair (Attwood et al., 2012); for example, for the proportionate-size goblets, the first participant poured the specified amount of liquid into the large and small goblets, whereas the next participant poured in reverse order (the small goblet first, followed by the large goblet). The color and lighting variables were randomly assigned during the experiment.

**Figure 2.**
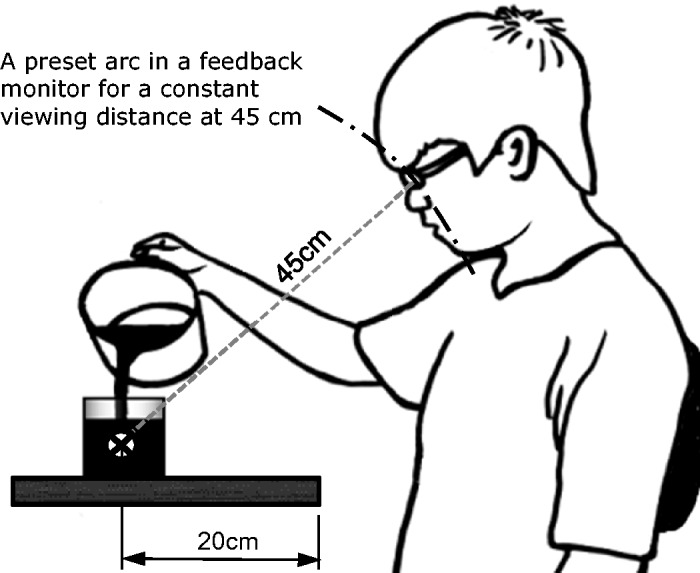
Schematic of the distance between a container and participant’s eyes in the liquid-pouring test.

The liquid-pouring experiment was based on, and revised from, the studies of Chen and Shi (2017) and Chen et al. (2017). To simulate the actual situation of a restaurant, the heights of the tables and chairs during the experiment were fixed. The participants adopted a natural seated posture throughout the experiment. To reduce the influence of distances on participant judgment, the distance between the center of the glass and the eyes of a participant remained constant at 45 cm. We employed a sagittal plane camera to monitor and correct the distance (Chen et al., 2017). To ensure this viewing distance, the experimenter verified that the participant eye positions matched a preset arc (Figure 2) in the feedback monitor. Because an actual restaurant situation was simulated in this study, a slight difference in the viewing angle among individuals was realistic and reasonable.

When the test began, the researcher first explained the experimental procedure in which participants poured a designated amount of water (100 or 200 ml) from a 700-ml bottle of cold water into specified pairs of glasses. Preceding the experiment, we had provided the participants with a round tumbler (100 ml) which was irrelevant to the experimental tumblers to practice for 5 minutes to become familiar with the perception of liquid volume (Chen et al., 2017). Considering that the participants might pour too much water for the glass, a pressure pipette was provided in the experiment to remove excess water for adjustment. After the experiment concluded, the researcher converted and recorded the amount into a capacity through weighing. We assumed that the density value of liquid used in the test was almost 1 (the same as water) and directly converted the weight into the poured volume. The red wine pouring followed the same experimental procedures.

### Statistical Analysis

This experiment collected data on the participant perceived amounts of water or red wine poured into pairs of goblets or tumblers as well as two types of geometric features (proportionate size and elongation) under two lighting levels (1,000 lux and 80 lux). The data were analyzed using a statistical software package (SPSS, version 22.0; SPSS, Chicago, IL) and the significance level (α) was set at .05. The effects of lighting, liquid color, and glass pairs on the visual capacity of participants with different container types and geometric features were examined using a three-way analysis of variance (ANOVA) without replication, and each participant was considered as a block. In addition, the ANOVA method was also used to analyze the effects of gender, lighting, and liquid color on volume perception for each individual glass.

## Results and Discussion

A three-way ANOVA was used to examine the participant volume perception of the four different container pairs to understand the effects of lighting, liquid color, and glass features on participant volume perception. In total, 1,920 data items were collected, and after outliers (those lying outside three standard deviations) were removed, the total number of valid samples was 1,844 with the proportion of valid data at 96%.

### Proportionate-Size Tumbler Pair

**Figure 3.**
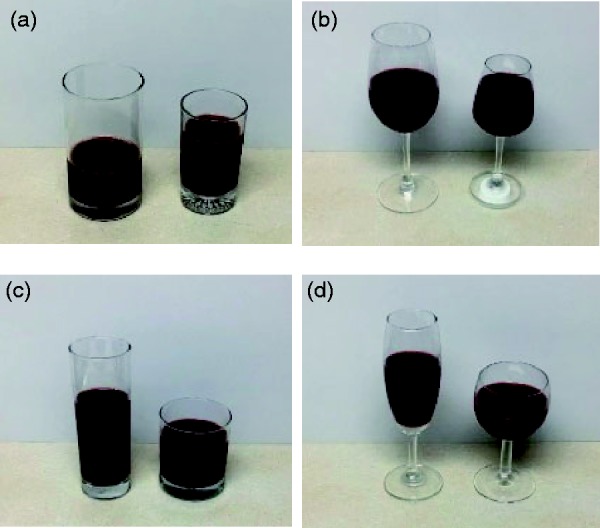
Visual cues for various container pairs into which the same assigned volumes were poured using red wine as an example. (a) Tumbler (proportionate size), (b) goblet (proportionate size), (c) tumbler (elongation), and (d) goblet (elongation).

Table 2 lists the three-way ANOVA results for the large and small tumblers, indicating that only proportionate size (i.e., large vs. small) had a significant effect on volume perception (*p* < .001). When asked to pour 100 ml of liquid into the tumblers, participants poured 126 ml and 99 ml into the large and small tumblers, respectively, with a difference of 27 ml; that is, the large tumbler had 26% more liquid. Kerr, Patterson, Koenen, and Greenfield (2009) also discovered that large tumblers received 26.5% more wine than did their smaller counterparts, a result that was similar to this study. Chen et al. (2017) found that with sufficient cues for glass height, participants poured approximately 16% more liquid in large tumblers than in the small ones. In general, people underestimated their volume perception in large tumblers regardless of the lighting (bright or dark) or drink color (light or dark). In practice, this may cause diners to unwittingly increase their intake amount (Figure 3(a)).

**Table 2.** Three-Way Analysis of Variance Results for the Proportionate-Size Tumblers and Goblets.

Sources	*df*	Tumbler proportionate-size pair			Goblet proportionate-size pair		
		*F*	*p*	Power	*F*	*p*	Power
Lighting (L)	1	2.212	.138	0.317	0.404	.525	0.097
Color (C)	1	1.187	.277	0.193	9.095	<.05	0.853
Size (S)	1	341.969	<.001	1.000	11.107	<.05	0.914
L × C	1	0.065	.798	0.057	0.341	.559	0.090
L × S	1	0.754	.386	0.139	0.099	.753	0.061
C × S	1	3.677	.056	0.481	0.054	.816	0.056
L × C × S	1	0.003	.957	0.050	0.010	.921	0.051

### Proportionate-Size Goblet Pair

Table 2 also exhibits the three-way ANOVA results for the large and small goblets, indicating that liquid color and proportionate size had significant effects (all *p* < .05) on volume perception. When averaged across other variables, the participant volume perception of water and red wine was 206 ml and 195 ml, respectively, with a difference of 11 ml, meaning that volume was underestimated when a transparent drink was poured into the goblet, whereas a red drink resulted in volume overestimation. The volume perception for large and small goblets was 194 ml and 207 ml, respectively, with a difference of 13 ml. A review of relevant literature demonstrated that the effects of goblet size on volume perception have not been systematically studied. In some of the few studies that have included goblets, Kerr et al. (2009) discovered a tendency to pour more liquid into large tumblers, whereas the same phenomenon was not observed with goblets. However, that study did not systematically manipulate the container size. Contrary to the tumbler analysis results, this study revealed that the large goblet volume was overestimated by participants, resulting in a smaller amount being poured. Chen et al. (2017) found that the effect of volume perception in relation to tumbler size was related to the participant viewing angle, with different viewing angles producing various visual cues that affected volume perception. In particular, when goblets (unlike tumblers) have no rim cues, the liquid height level may become an essential cue for volume perception (Figure 3(b)). Attwood et al. (2012) discovered that irregular shapes increased the difficulty of judging perception. Troy et al. (2018) also demonstrated that pouring in tulip and curved glasses was less accurate than in straight glasses. The test results for the proportionate-size goblet pairs may have varied from those of tumblers because the viewing angle and visual cues were more challenging.

### Elongation Tumbler Pair

Table 3 presents the three-way ANOVA results for tall and slender versus short and wide tumblers, demonstrating that the three main effects reached statistical significance (all *p* < .05). Participant volume perception was affected by lighting; the pouring amount under dark conditions was significantly greater at 186 ml compared with 181 ml under bright conditions. The volume perception of water and red wine was 187 and 179 ml, respectively, and 197 and 169 ml for the tall and slender tumblers versus short and wide, respectively. The difference between actual and target pouring amounts indicated that volume perception for the short, wide tumbler varied more; the volume perception for the short, wide tumbler was 31 ml lower than the target amount of 200 ml, with an overestimation of approximately 15.5%, compared with the tall, slender tumbler in which the pouring amount of 197 ml neared the target amount. This result was consistent with that of Chen and Shi (2017) but contradicted the study of Wansink and Van Ittersum (2003). Chen et al. (2017) discovered that when volume perception was guided by the glass rim cue, the amount poured in the short, wide tumbler was lower than that in the tall, slender tumbler; that is, the volume of the short, wide tumbler was overestimated (Figure 3(c)). By contrast, the results of Wansink and Van Ittersum (2003) were based on the vertical–horizontal illusion of the tumbler height. Based on these results, pouring dark red drinks (e.g., red wine) in a bright environment into a short, wide tumbler may result in overestimating the liquid volume and thereby reducing the intake amount. Therefore, the suggestion of Wansink and Van Ittersum (2003) that using tall, slender glasses would reduce intake amounts may not be accurate for all situations.

**Table 3.** Three-Way Analysis of Variance Results for the Elongation-Group Tumblers and Goblets.

Sources	*df*	Tumbler elongation pair			Goblet elongation pair		
		*F*	*p*	Power	*F*	*p*	Power
Lighting (L)	1	3.992	<.05	0.514	1.236	.267	0.199
Color (C)	1	8.675	<.01	0.836	2.494	.115	0.351
Elongation (E)	1	104.268	<.001	1.000	32.776	<.001	1.000
L × C	1	0.780	.377	0.143	0.119	.731	0.064
L × E	1	0.372	.542	0.093	0.314	.575	0.087
C × E	1	0.809	.369	0.146	0.217	.642	0.075
L × C × E	1	0.001	.971	0.050	0.603	.438	0.121

### Elongation Goblet Pair

Table 3 also presents the three-way ANOVA results for tall and slender versus short and wide goblets; only the elongation showed significant effects (*p* < .001). The participant volume perception when using tall and slender versus short and wide goblets was 113 ml and 102 ml, respectively. Compared with a target amount of 100 ml, the tall, slender goblet caused participants to underestimate the volume and resulted in an overage of 13 ml; the volume perception for the short, wide goblet was nearer to the target. Similarly, Chen and Shi (2017) discovered that the liquid in a tall, slender goblet surpassed the target volume by approximately 12 ml. A possible reason for this perception variance is that participants may have been influenced by the rim diameter cue (Figure 3(d)).

### Gender Differences

**Figure 4.**
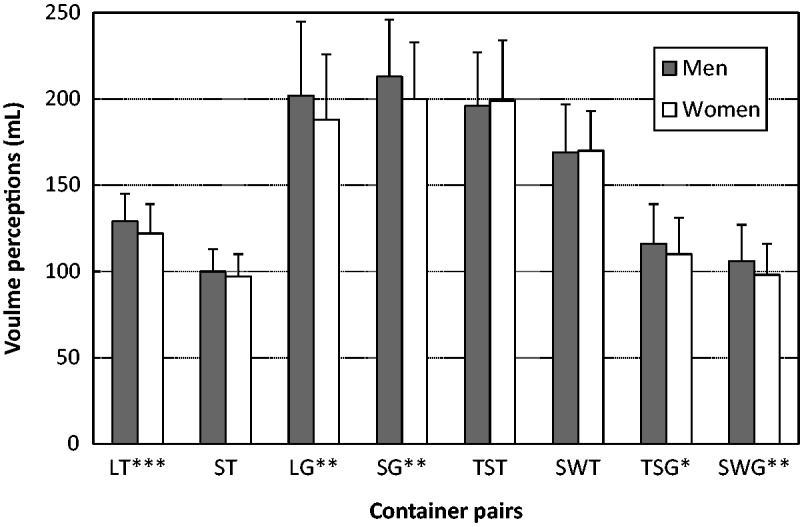
Gender differences in volume perception regarding each individual container (*p < .05, **p < .01, and ***p < .001). LG = large goblet; SG = small goblet; LT = large tumbler; ST = small tumbler; TSG = tall, slender goblet; SWG = short, wide goblet; TST = tall, slender tumbler; SWT = short, wide tumbler.

In this study, a three-way ANOVA was also employed to examine the differences in volume perception for each individual container. However, when compared between the sexes, lighting and liquid color variables exhibited relatively little influence on volume perception. When averaged across other variables among the eight containers, a gender difference was observed in the volume perception of all goblets, whereas no gender difference was observed in all but the large tumbler (Table 4). Attwood et al. (2012) discovered that irregular shapes increased the difficulty of judging volume perception, an observation that was also reflected in gender-related factors. Figure 4 reveals that in all the significant gender differences related to volume perception, men always poured more than women did; that is, men tended to underestimate the container volume. The volume perception of different target volumes (100 ml or 200 ml) was also complicated by both gender and glass type factors. In addition, all participants substantially overestimated the volume of the short, wide tumbler, resulting in a small amount being poured. These observations require clarification in further studies.

**Table 4.** Effects of Gender on Volume Perception.

Variables	Containers	Characteristics	*F*	*p*	Power
Proportionate size	Tumbler	Large	10.558	<.001	0.899
		Small	2.047	.154	0.297
	Goblet	Large	6.848	<.01	0.741
		Small	7.739	<.01	0.791
Elongation	Tumbler	Tall, slender	0.350	.555	0.091
		Short, wide	0.074	.785	0.058
	Goblet	Tall, slender	4.152	<.05	0.528
		Short, wide	7.747	<.01	0.791

### Study Summary

Table 5 summarizes the significant effects of each variable on volume perception of the four container pairs. Volume perception was significantly affected by the geometric features (i.e., proportionate size and elongation) of all glass pairs. Some results matched those of relevant studies, whereas some were contradictory. A possible reason for this disparity is that volume perception was mainly affected by the differences in visual cues (i.e., rim diameter or height) and glass shapes. Therefore, using a single glass type to assess participant variant estimation may be too simplistic. In addition, environmental lighting and liquid color inconsistently affected volume perception, which may have been concurrently influenced by the different glass types and geometric features. However, other factors that were not examined in this study may have also affected the susceptibility to optical illusion, such as food deprivation (Zitron-Emanuel & Ganel, 2018).

**Table 5.** Summary of the Significant Effects of Each Variable on Volume Perception.

	Tumbler pairs			Goblet pairs		
Variables	Lighting	Color	Proportionate size	Lighting	Color	Proportionate size
*p*	–	–	<.001	–	<.05	<.05
Variables	Lighting	Color	Elongation	Lighting	Color	Elongation
*p*	<.05	<.01	<.001	–	–	<.001

## Conclusion

In this study, a water and red wine-pouring experiment was conducted to understand the effects of environmental lighting and liquid color on the volume perception of geometric features of different glass types. The results demonstrated that the goblet and tumbler pairs in both the proportionate-size and elongation groups produced significant effects on volume perception, whereas the effects of lighting and liquid color were only observed in specific experimental groups. This study also revealed that the volume perception of men was generally underestimated compared with that of women, and the men poured more water and wine. Both men and women overestimated volume perception of the short, wide tumbler (by approximately 15%). Therefore, a 200-ml short, wide tumbler may be recommended for individuals who wish to reduce their intake of alcohol (e.g., to ameliorate alcohol abuse) or other beverages because the volume of 200-ml glasses appears to be visually satisfying.
